# Toward Achieving Rapid Estimation of Vitamin C in Citrus Peels by NIR Spectra Coupled with a Linear Algorithm

**DOI:** 10.3390/molecules28041681

**Published:** 2023-02-09

**Authors:** Weiqing Zhang, Mei Lin, Hongju He, Yuling Wang, Jingru Wang, Hongjie Liu

**Affiliations:** 1 Zhejiang Citrus Research Institute, Zhejiang Academy of Agricultural Sciences, Taizhou 318026, China; 2 School of Food Science, Henan Institute of Science and Technology, Xinxiang 453003, China; 3 School of Life Science & Technology, Henan Institute of Science and Technology, Xinxiang 453003, China; 4 School of Chemistry and Chemical Engineering, Guangxi University, Nanning 530004, China

**Keywords:** vitamin C, citrus peel, near-infrared (NIR), determination, linear algorithm

## Abstract

Citrus peels are rich in bioactive compounds such as vitamin C and extraction of vitamin C is a good strategy for citrus peel recycling. It is essential to evaluate the levels of vitamin C in citrus peels before reuse. In this study, a near-infrared (NIR)-based method was proposed to quantify the vitamin C content of citrus peels in a rapid way. The spectra of 249 citrus peels in the 912–1667 nm range were acquired, preprocessed, and then related to measured vitamin C values using the linear partial least squares (PLS) algorithm, indicating that normalization correction (NC) was more suitable for spectral preprocessing and NC-PLS model built with full NC spectra (375 wavelengths) showed a better performance in predicting vitamin C. To accelerate the predictive process, wavelength selection was conducted, and 15 optimal wavelengths were finally selected from NC spectra using the stepwise regression (SR) method, to predict vitamin C using the multiple linear regression (MLR) algorithm. The results showed that SR-NC-MLR model had the best predictive ability with correlation coefficients (*r*_P_) of 0.949 and root mean square error (RMSE_P_) of 14.814 mg/100 mg in prediction set, comparable to the NC-PLS model in predicting vitamin C. External validation was implemented using 40 independent citrus peels samples to validate the suitability of the SR-NC-MLR model, obtaining a good correlation (R^2^ = 0.9558) between predicted and measured vitamin C contents. In conclusion, it was reasonable and feasible to achieve the rapid estimation of vitamin C in citrus peels using NIR spectra coupled with MLR algorithm.

## 1. Introduction

Citrus fruit including oranges, tangerines, mandarins, clementines, grapefruit, pomelos, lemons, limes, and other minor varieties, originated in Southeast Asia, have been cultivated for the last 4000 years [1], and are some of the most popular fruits in the world. According to the statistics from Food and Agriculture Organization (FAO), citrus fruit are widely planted in more than 140 countries worldwide, with an annual output of about 100 million tons (Asia and the Americas accounts for over 70%) every year, playing an important role in the world’s fruit trade and economy. In Asia, China is the largest producer and exporter of citrus fruit, with annual yields of over 40 million tons, exceeding 20% of the world’s total fruit production.

Citrus fruit are mainly used to produce fresh juices or citrus-based drinks or as fresh foods for direct consumption of their edible parts, and large amounts of byproduct wastes are always generated either during industrial processing or after eating, such as citrus peels [2], which can cause environmental pollution issues. 

Several studies have been reported that show that citrus peels are rich in various nutrients and bioactive ingredients, including carbohydrates, vitamin C, carotene, polyphenols, flavonoids, essential oils, pectin, etc. [3], which make citrus peels a good material for producing value-added products such as additives, antioxidants, biological enzymes, biofuels, nutritional supplements, etc. [4]. For examples, citrus peel oils and peel extracts were analyzed and evaluated as potential food additives for preventing cancer and as anticancer agents [5]. Methanolic extracts of citrus peels showed strong antioxidant activities in terms of free fatty acid content, peroxide value, and iodine value, which was almost same good as synthetic antioxidants such as butylated hydroxy anisole (BHA) and butylated hydroxy toluene (BHT) [6]. Citrus peels (orange, mandarin, lemon, grapefruit) were hydrolyzed to produce bacterial celluloses (BCs), and the obvious characteristics of great water holding capacity, thermal stability, crystallinity, and thin fiber diameter were proved in the produced BCs [7]. Dried citrus peels were also used as substrates for producing pectinase by *Aspergillus niger*, obtaining maximum enzyme activity when 15% substrates involved [8]. Sweet lime peel and orange peel were characterized with a good amount of carbon, hydrogen, and oxygen, and that indicated the good potential of citrus peel in ethanol production [9]. 

Vitamin C, also known as L-ascorbic acid, is one of important biologically active compounds and has been found to have higher amounts in citrus peels than in pulp and seeds [10]. Vitamin C has a great antioxidant activity and can be used as a high efficiency antioxidant to scavenge active oxygen groups and some free radicals such as superoxide, singlet oxygen, hydrogen peroxide and hydroxyl radical [11]. Vitamin C is regarded as a good dietary supplement exhibiting functions of both pro-oxidant and antioxidant [12]. The levels of vitamin C can regulate the functions of blood-forming stem cells and the development of leukemia [13]. Using citrus peels as raw materials to extract vitamin C is one of good strategies for peel reuse and has been proved to be reasonable and feasible [14,15]. It is better and very necessary to evaluate the contents of vitamin C in citrus peels before recycling, as the concentration of vitamin C varies in different varieties of citrus peels [10]. 

However, traditional methods for determining vitamin C in foods are mainly chemical assays such as official dichloroindophenol titrimetry [16], high-performance liquid chromatography (HPLC) and HPLC-based methods [17], ultra-performance liquid chromatography (UPLC) and UPLC-based methods [18,19], and electrochemical methods [20]. Some physical approaches are proposed to determine the vitamin C in foodstuffs, such as the calorimetric method [21] and cyclic voltammetry [22]. The feasibility of enzymatic methods applied in vitamin C determination has also been demonstrated [23]. Although with these methods, vitamin C can be determined with a low limit of detection, high-precision, specificity and recovery, i.e., good accuracy and reliability, are time-consuming, tedious, laborious, destructive and requires well-trained staff to operate machines. Moreover, it is very difficult to achieve real-time rapid and nondestructive evaluation of vitamin C when large numbers of samples are required for detection. A novel method is necessary and should be developed to determine vitamin C in a rapid way. On the other hand, vitamin C is vulnerable to oxidation [24], which also needs a rapid method to evaluate the levels of vitamin C, helping to use citrus peels in a timely manner. 

Near-infrared (NIR) spectroscopy, an optical technology, has the obvious advantages of little or no sample pretreatment, non-pollution, rapidness, non-destructive inspection, simultaneous detection of multiple components, and can be potentially applied for industrial purposes [25]. By absorbing electromagnetic waves in the 780–2526 nm range, NIR provides information from molecular vibrations and combined overtones of chemical groups such as C-H, O-H, N-H and S-H, which can be analyzed by chemometrics to relate to target parameters for qualitative and quantitative evaluation [26], i.e., model establishment between NIR spectra and target parameters. NIR technology has been used for the quality evaluation in various foods in terms of physical, chemical, nutritional, textural and microbial properties, with good or satisfied and even excellent performance, indicating a great potential of NIR in food industrial application [27,28]. However, few studies on NIR technology for the quality evaluation of fruit peels have been reported, especially vitamin C in citrus peels. 

In this study, we proposed to develop a NIR-based method for the rapid assessment of vitamin C levels in citrus peels. It is also the first effort to investigate the potential of NIR technology in monitoring the vitamin C of citrus peels. This study will provide a novel technical support to facilitate the use of citrus peels, reducing the waste. 

## 2. Results and Discussion

### 2.1. Statistical Values of Vitamin C 

**Figure 1:**
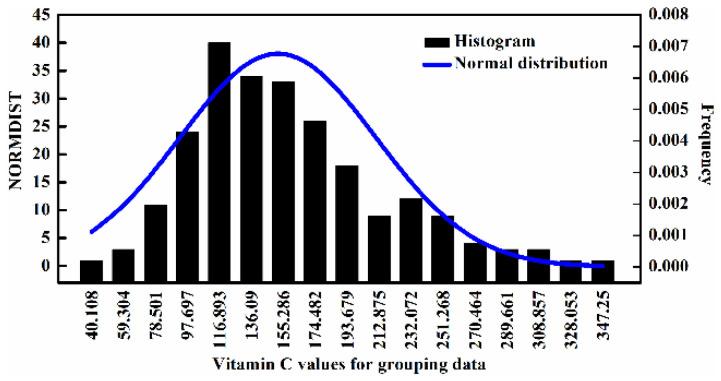
Normal distribution of measured vitamin C values.

A total of 249 citrus peels samples from 50 different varieties were prepared and their vitamin C contents were measured and arranged from small to large, and one of every three values was selected for model prediction, and the remaining values were used for model calibration and cross-validation. The statistical details are shown in Table 1. After calculation, it was found that all the measured vitamin C values obeyed normal distribution, indicating the statistical significance. On the other hand, the subsequent *F*-test and *t*-test required all the measure data to meet normal distribution. The specific data distribution is shown in Figure 1.

### 2.2. Spectral Profiles of Samples

**Figure 2:**
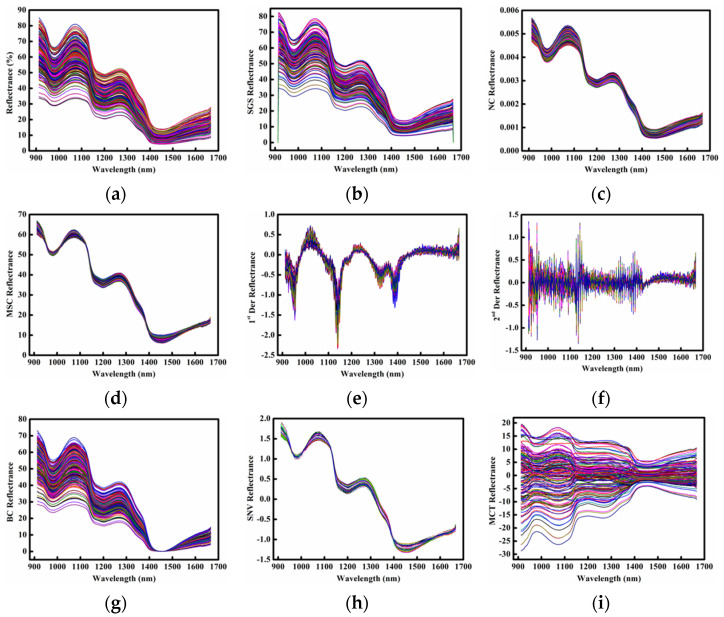
Spectral characteristics of citrus peel samples (249 curves with different colors). (**a**) Raw spectra; (**b**) Savitzky−Golay smoothing (SGS) spectra; (**c**) normalization correction (NC) spectra; (**d**) multiple scattering correction (MSC) spectra; (**e**) 1st derivative (1st Der) spectra; (**f**) 2nd derivative (2nd Der) spectra; (**g**) baseline correction (BC) spectra; (**h**) standard normal variate (SNV) spectra; (**i**) mean centering transformation (MCT) spectra.

The extracted mean raw and preprocessed spectral profiles of citrus peel samples in the range of 912 t 1667 nm are shown in Figure 2. Three absorption peaks at around 980 nm, 1200 nm, and 1450 nm were observed and originated from second overtones O-H stretching vibration (water absorption), second overtone C-H stretching (fat absorption) and first overtones O-H stretching vibration (water absorption), respectively [29]. 

It was observed that the positions of absorption peaks (refer to the vertical distance from the x-coordinate) for the 249 curves in Figure 2a were different, and that was probably due to the difference of physicochemical components in each citrus peel sample. After spectral preprocessing, the changes in absorption peak position in different each plots were also observed, which may be due to the elimination of interference information from raw spectra such as electrical noise, light scattering and baseline drift, etc. 

Although the specific absorption peak of vitamin C was not found, the relevant useful NIR spectra can be mined and extracted by applying appropriate chemometrics, to relate to the vitamin C contents that is modeling, achieving the quantitatively prediction of vitamin C in citrus peels. 

### 2.3. Predicting Vitamin C Using Full Wavelength

The full-band spectra (raw and preprocessed) within the range of 912 to 1667 nm (375 wavelengths) were mined to related to the measured vitamin C by partial least squares (PLS) algorithm, resulting in different performance of the nine PLS models in prediction of vitamin C of citrus peels samples, with correlation coefficients of prediction (*r*_P_) of 0.877–0.974 and root mean square error of prediction (RMSE_P_) of 10.671–23.916 mg/100 g (Table 2). 

It was also observed that the PLS models built with eight preprocessed spectra had different predictive abilities in predicting vitamin C, compared with the RAW-PLS model using raw spectra. Among, the NC-PLS model built with NC spectra showed the best performance in predicting vitamin C content of citrus peels samples (*r*_P_ = 0.956, RMSE_P_ = 10.671 mg/100 g, residual predictive deviation (RPD) = 4.189), although largest numbers of latent variables (LV) involved, which indicated that NC was more suitable for preprocessing the spectra of 912–1667 nm and that was probably due to the elimination of adverse effects caused by outlier samples. Moreover, the NC-PLS model performed better than the RAW-PLS model, in terms of correlation coefficients (*r*), root mean square errors (RMSEs), RPD, absolute value of difference between RMSE_P_ and root mean square error of calibration (RMSE_C_) (ΔE) values, indicating that implement of spectra pretreatment by NC method indeed improved the predictive accuracy and precision of the RAW-PLS model. In general, spectral preprocessing was necessary to predict the vitamin C of citrus peels, and an appropriate preprocessing method such as NC was required. 

There are some reports on NIR technology in prediction of vitamin C concentration in other fruit such as tomatoes (range: 1295–2611 nm, *r*_P_ = 0.81, RMSE_P_ = 4.09 mg/100 g), oranges (range: 4000–10,000 cm^−1^, *r*_P_ = 0.71, RMSE_P_ = 94.9 mg/L), acerola (range: 800–2500 nm, *r*_P_ = 0.99, RMSE_P_ = 166.27 mg/100 g), and apple (range: 4000–10,000 cm^−1^, *r*_P_ = 0.917, RMSE_P_ = 4.8 mg/100 g) [30], different from our study, which was probably due to the different spectral ranges and samples involved in modeling. Until now, this is the first time using NIR to predict vitamin C level in citrus peels, and the satisfactory results were obtained. The further wavelength selection and model optimization were performed based on the NC spectra.

### 2.4. Optimal Wavelengths Selected by Four Different Methods

**Figure 3:**
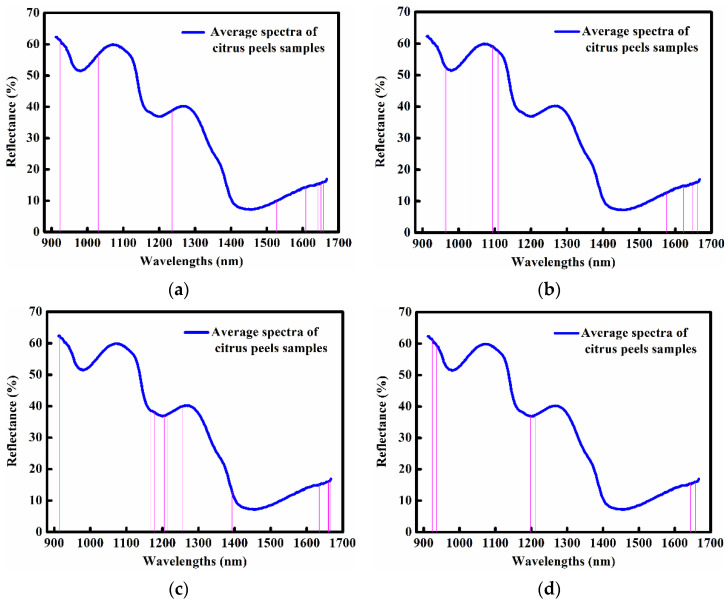
Specific locations of selected optimal wavelengths. (**a**) RC method; (**b**) SR method; (**c**) SPA method; (**d**) CARS method.

The optimal wavelengths were selected from NC spectra by regression coefficients (RC), stepwise regression (SR), successive projections algorithm (SPA) and competitive adaptive reweighted sampling (CARS) methods, respectively, and the results are shown in Table 3. After wavelength selection, the wavelength number decreased from 375 to 5–22, with the wavelength reduction of over 94%. It was also observed that most of the selected optimal wavelengths mainly located in the three regions of 912–1030 nm, 1161–1255 nm, and 1576–1667 nm, which are shown in Figure 3 and indicated that more spectral information related to vitamin C prediction existed in these regions.

### 2.5. Predicting Vitamin C Using Optimal Wavelengths

Based on the selected optimal wavelengths, the NC-PLS model was optimized and four optimized PLS models (RC-NC-PLS, SR-NC-PLS, SPA-NC-PLS, CARS-NC-PLS) were respectively established and their performance in predicting vitamin C of citrus peels samples are shown in Table 4. 

Among these, the SR-NC-PLS model built with 15 optimal wavelengths selected from NC spectra by SR method showed a good predictive ability, with higher values of *r*_P_ (0.936) and RPD (3.482) as well as lower values of RMSE_P_ (16.689 mg/100 g) and ΔE (1.257 mg/100 g), better than those of other three optimized PLS models, which was probably due to the different optimal wavelengths involved in model construction. The results also indicated that the SR method was the best option to select optimal wavelengths. 

In fact, multiple linear regression (MLR) can also be used for predicting a target parameter of samples in the situation where the number of wavelengths is less than the number of samples [31]. MLR is a classic linear algorithm and works to interpret linear relationship between one dependent variable and two or more independent variables [32]. In this study, based on the same selected optimal wavelengths, four MLR models including RC-NC-MLR, SR-NC-MLR, SPA-NC-MLR, and CARS-NC-MLR were respectively developed and assessed in terms of *r* and RMSE values. It was found that the SR-NC-MLR model had the best performance in predicting vitamin C among the four MLR models, carrying the largest values of *r*_P_ (0.949) and RPD (4.260) as well as smallest values of RMSE_P_ (14.814 mg/100 g) and ΔE (1.384 mg/100 g), better than the SR-NC-PLS model. 

In addition, through a comparative analysis, it was found that the SR-NC-MLR model was comparable to the NC-PLS model in predicting vitamin C of citrus peels samples, indicating that the optimization of NC-PLS model was successful. This also meant that the selected 15 optimal wavelengths and the full 375 wavelengths contributed similarly to the vitamin C prediction. 

### 2.6. F-Test and T-Test Analysis

As shown in Table 5, after implementing *F*-test, it was found that the *F* value (1.004) was smaller than the *F* (one-tailed critical value) value for the SR-NC-MLR model, which indicated that no significant difference between the measured value and predicted value of vitamin C contents in citrus peels existed.

It was also observed from *t*-test results that the *t* value was less than the *t* (two-tailed critical value) value, revealing that there was no significant difference between the mean values of the measured value and the predicted value of vitamin C contents in citrus peels. 

In short, the good soundness and predictive validity of the SR-NC-MLR model were verified using the *F*-test and *t*-test analysis. In other words, it was reasonable and feasible to apply the SR-NC-MLR model to predict vitamin C contents of citrus peels.

### 2.7. Independent External Validation of Best Optimized Model

**Figure 4:**
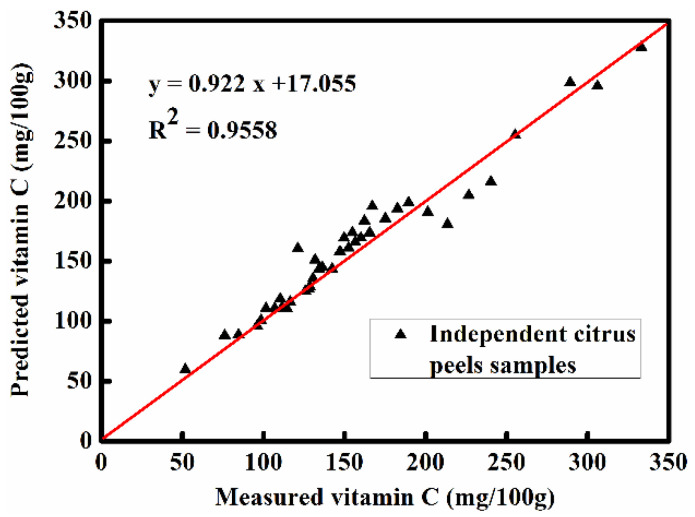
Predicted and measured values of vitamin C contents.

Forty citrus peels were used as independent samples to externally validate the validity and suitability of the SR-NC-MLR model in predicting vitamin C contents, and the results are shown in Figure 4. A good correlation (R^2^ = 0.9558) was found between the predicted and the measured values of vitamin C contents, indicating the good predictive performance of the SR-NC-MLR model. 

## 3. Materials and Methods

### 3.1. Preparation of Citrus Peel Samples 

Two hundred and forty-nine fresh intact and undamaged citrus fruit (50 varieties and five of each (four of the Xuegan variety): Judaro, Miyagawa wase, Nanfengmiju, Nankou,,Kaixuangan, Red Sene, Clementine, Amakusa, Owari, Yura Wase, Taguchi wase, Cocktail grapefruit, Bendizao, Huyou, Hongyugan, Ueno Wase, Hongmeiren, Aoshima, Ougan, Green Ougan, Ponkan, Navel orange, 439, Honeybelle tangelo, Himekoharu, 60, Okitsu No.60, Lime, Sweet Spring, Gaocheng, Flame grapefruit, Valencia orange, Gonggan, Seihou, Chachigan, Huyou, Asumi, Washington Sanguine blood orange, Tarocco blood orange, Xuegan, Jincheng, Gaotaocheng, 123-1, Tsunokaori Tangor, Murcott, Manju, Moro blood orange, Haruka, Orah, Liubencheng, Sokitsu) were randomly selected after harvesting from the Citrus Breeding Base of Zhejiang Citrus Research Institute, Taizhou City, Zhejiang Province, China and immediately transported to the laboratory to be stored at cold temperature (4 ± 0.5 °C) for further spectral acquisition and vitamin C determination. Before the test, the peel surface was examined carefully again, and a simple cleaning was performed to guarantee minimal impact on further spectral collection.

### 3.2. Spectral Collection and Preprocessing

A portable NIR spectroscopy device (Isuzu Optics Corp., Zhubei, Taiwan) was used to collect spectral information of citrus peels in reflectance mode. The machine mainly consists of four sections including a spectrograph (covering spectral range of 900–1700 nm, 1 mm InGaAs detector), a ring-shaped Halogen lamp (20 W), a glass plate (diameter, 60 mm; height, 10 mm). and spectral analysis software (NIRez 2.0 Rice, Isuzu Optics Corp., Taiwan). The device was operated by setting the exposure time of 0.63 ms and the scan number of 5. Before the spectral collection of samples, the device was calibrated by scanning a white tile bar with reflectance of 99.99% and then turning off the light source to ensure 0.00% reflectance. 

Before each test, several citrus fruit were taken out and one piece of peel with size of 20 mm × 20 mm (length × width) from each citrus fruit was cut to put into the glass plate of NIR device. Each citrus peel was scanned five times to obtain the average spectra. Finally, a total of 249 spectra of citrus peel samples were prepared for further analysis. The spectra in the range of 912–1667 nm (375 wavelengths) was only considered and analyzed, because of obvious noises existed in the two regions of 900–912 nm and 1667–1700 nm. 

The process of spectral collection is always negatively influenced by several factors such as sample status, light scattering, stray light, baseline drift, instrument response and the surrounding environment [33]. Therefore, it is quite necessary to perform spectra preprocessing to minimize or even eliminate the undesirable effects, improving the signal-to-noise ratio of spectra and predictive ability of subsequent constructed model. In this study, six preprocessing methods including SGS, NC, MSC, 1st Der, 2nd Der, BC, SNV, and MCT were applied to preprocess the collected raw spectra, respectively. 

SGS uses polynomials to achieve data smoothing, based on the PLS algorithm, retaining useful information in signal analysis and eliminating random noise [34]. NC is used to eliminate influence of changes in optical path or sample dilution on spectra [35]. MSC can eliminate noises caused by specular reflection and non-uniformity of sample, spectral baseline drift and non-repeatability [36]. Derivation is an effective preprocessing method used to eliminate baseline drift and improve spectral resolution. The 1st Der and 2nd Der can remove the constant baseline and the first functional baseline, respectively [37]. BC can effectively correct drifts originated from electronic offset, dark current and readout noise [38]. SNV is applied to reduce influences of uneven particle size and non-specific scattering of particle surface [39]. MCT is realized using sample spectra minus mean spectra of calibration set to increase the difference between sample spectra, thus improving robustness and prediction ability of model [40]. 

All the spectral preprocessing were completed using software Unscramble 10.3X (CAMO, Oslo, Norway). 

### 3.3. Measurement of Vitamin C 

The vitamin C contents in citrus peel samples were determined using the chemical 2-6-dichlorophenol indophenol titration method (AOAC Method 967.21) [41], three times for each sample, and the averaged values were used. In this study, the NIR-based method was developed and compared with the official chemical method, expecting to potentially substitute the official method for vitamin C determination in the future. 

### 3.4. Quantitative Relationship Establishment between Spectra and Vitamin C 

The quantitative relationships between the raw, preprocessed NIR spectra and the measured vitamin C values were respectively established by applying linear PLS regression algorithm. PLS is always used to build the fundamental relationship between two matrixes (*X* and *Y*), explaining *Y* space with greatest variance by finding multidimensional directions in *X* space, and is particularly suitable when *X* matrix (predicted) has more variables than *Y* matrix (observed), as well as when there is multicollinearity in *X* [42]. PLS combines the advantages of principal component analysis (PCA), canonical correlation analysis (CCA) and multiple linear regression (MLR) analysis, and can achieve predictive function through extracting a group of irrelevant latent variables (LV) [43]. PLS model performance is related to the number of LV, and a good PLS model always has small number of LV [44]. 

The predictive performance of the PLS model is evaluated mainly using *r* and RMSE in the calibration set (*r*_C_ & RMSE_C_), cross-validation set (*r*_CV_ & RMSE_CV_), and prediction set (*r*_P_ & RMSE_P_) [45]. The cross-validation is implemented by leaving one sample out from the calibration set in turn, and then rebuilding a model with the remaining samples to predict the excluded sample, i.e., leave-one-out cross-validation [46]. Generally, a PLS model with good performance always have higher values of *r* and lower values of RMSEs. Two other parameters including RPD and ΔE are also used to assess the PLS model quality. RPD is ratio of standard deviation of measured values to RMSE_P_ values in prediction set. ΔE value is used to indicate the model robustness. A good PLS model is usually accompanied by a greater value of RPD and a smaller value of ΔE [47].

In this study, by inputting a matrix (375 NIR spectra as *X* variables, 166 vitamin C values as *Y* variables) to run PLS algorithm and using the remaining 83 vitamin C values for prediction purpose, an intrinsic relation between the two variables was explored, i.e., PLS modeling. The established PLS model was evaluated by terms of *r*_C_, *r*_CV_, *r*_P_, RMSE_C_, RMSE_CV_, RMSE_P_, RPD and ΔE. 

### 3.5. Optimal Wavelength Selection and Model Optimization 

Generally, NIR spectral analysis technique is accompanied by a large amount of spectral data, which inevitably contains some spectral variables carrying noise, non-information wavelength or even interference information, resulting in the decrease of predictive efficiency [48]. Selection of wavelengths holding useful information is therefore very necessary and has been a key step in NIR data analysis, which can greatly reduce the data calculation, accelerate the prediction, improve the model prediction accuracy, and effectively prevent the overfitting prediction [49]. 

Four efficient methods including RC, SR, SPA, and CARS were respectively used to select the optimal wavelengths for further model optimization. In the procedure of RC, the wavelengths with large absolute values of regression coefficients in developed PLS model were selected as optimal wavelengths [50]. By running SR program, the optimal wavelengths were automatically selected by repeating the operations of forward addition and reverse deletion of spectral variables at the same time, and terminating with the minimum values of residual sum of squares (MVSSS) via increasing spectral variables [51]. In SPA process, by sequentially executing the selection of candidate subsets by projection, the evaluation of candidate subsets by predicted residual error sum of squares (PRESS) and the elimination of variables through *F*-test criterion, the wavelengths corresponding the minimum number and the lowest values of PRESS were considered to be optimal wavelengths [52]. In the CARS method, the important wavelengths were picked out by assessing the importance of each wavelength through the corresponding absolute value of regression coefficient, according to the law of survival of the fittest [53].

Using the same modeling process, the optimized PLS model was developed by inputting a new matrix containing the selected optimal wavelengths as *X* variables and 166 vitamin C values as *Y* variables. The remaining 83 vitamin C values were used for prediction. The optimized PLS model was also evaluated using the same parameters mentioned above. 

The RC process and model optimization were performed in software Unscrambler 10.3X (CAMO, Oslo, Norway). The SR, SPA and CARS program were executed in software Matlab R2018a (The Mathworks, Inc., Natick, MA, USA). 

### 3.6. Statistical Two-Sample Analysis

*F*-test and *t*-test two-sample analysis were conducted to verify the suitability of the established model in predicting vitamin C of citrus peels, ensuring the model soundness and predictive reliability. *F*-test, also called homogeneity test of variance, is a test under null hypothesis with statistical values obeyed *F*-distribution [54]. The *t*-test, also called Student’s *t*-test, is applicable in three simultaneously required conditions of two sets of samples coming from the normal population, independence of the two sets of samples and satisfying homogeneity of variance (pass *F*-test) [55]. *F*-test was applied to test whether there is a significant difference between the variances of the measured and predicted vitamin C values. *t*-test was conducted to examine whether there is significant difference between the mean values of the measured and predicted vitamin C values. *F*-test was completed before the *t*-test.

### 3.7. External Validation of Model

To further evaluate the applicability and validity of the established calibration model, it is necessary to conduct external validation using a set of independent samples. For achieving the stable and effective prediction of vitamin C, 40 independent citrus peel samples were randomly collected from fresh harvested citrus fruit and used to validate the best optimized PLS model externally. The operation of the external validation was executed in software Unscramble 10.3X (CAMO, Oslo, Norway). 

## 4. Conclusions

This study aimed to investigate the potential of NIR (912–1667 nm) combined with linear algorithms to determine vitamin C contents in citrus peels. NC method was more appropriate to preprocess the raw NIR spectra, and NC-PLS model constructed with full NC spectra showed a better performance in predicting vitamin C contents (*r*_P_ = 0.956, RMSE_P_ = 13.798 mg/100 g). Fifteen optimal wavelengths (915, 927, 960, 965, 1016, 1028, 1094, 1109, 1397, 1576, 1623, 1642, 1648, 1662, and 1664 nm) were further selected from NC spectra by SR method and applied to optimize the full band NC-PLS model through MLR algorithm, resulting in an optimized SR-NC-MLR model with similar good predictive abilities in predicting vitamin C contents, with *r*_P_ of 0.949 and RMSE_P_ of 14.814 mg/100 g, compared with the NC-PLS model. The reasonability and feasibility of the SR-NC-MLR model was verified by means of *F-*test and *t*-test analysis. The validity and suitability of SR-NC-MLR model was further verified using 40 independent citrus peel samples. It concluded that the developed NIR-based method is simple, efficient, and can be used for rapid determination of vitamin C content in citrus peels to facilitate peel recycling. 

## Figures and Tables

**Table 1:** Statistical vitamin C values (mg/100 g) determined by official method.

Sample Set	Number of Sample	Minimum	Maximum	Mean	Standard Deviation
Calibration set	166	40.108	342.413	152.379	59.085
Prediction set	83	43.304	333.110	151.582	58.106

**Table 2:** Prediction of vitamin C (mg/100 g) in citrus peel by PLS model using full 375 wavelengths.

Spectra	Model	Number of LV	Calibration		Cross-Validation		Prediction			ΔE
			*r*_C_	RMSE_C_	*r*_CV_	RMSE_CV_	*r*_P_	RMSE_P_	RPD	
RAW	RAW-PLS ^1^	13	0.957	13.077	0.930	15.703	0.928	16.343	3.537	3.266
SGS	SGS-PLS ^2^	14	0.971	11.314	0.934	15.235	0.926	16.319	3.542	5.005
NC	NC-PLS ^3^	14	0.974	10.674	0.953	13.174	0.956	13.798	4.189	3.124
MSC	MSC-PLS ^4^	14	0.974	10.671	0.932	15.499	0.918	16.428	3.518	5.757
1st Der	1st Der-PLS ^5^	8	0.944	13.170	0.889	17.907	0.877	18.114	3.191	4.944
2nd Der	2nd Der-PLS ^6^	5	0.915	18.949	0.892	21.566	0.881	22.335	2.588	3.386
BC	BC-PLS ^7^	12	0.950	14.237	0.906	20.135	0.913	20.253	2.854	6.016
SNV	SNV-PLS ^8^	11	0.947	14.611	0.894	21.421	0.887	23.916	2.417	9.305
MCT	MCT-PLS ^9^	13	0.957	13.077	0.910	20.703	0.893	21.744	2.658	8.667

^1^ PLS model built with raw spectra; ^2^ PLS model built with SGS spectra; ^3^ PLS model built with NC spectra; ^4^ PLS model built with MSC spectra; ^5^ PLS model built with 1st Der spectra; ^6^ PLS model built with 2nd Der spectra; ^7^ PLS model built with BC spectra; ^8^ PLS model built with SNV spectra; ^9^ PLS model built with MCT spectra.

**Table 3:** Optimal wavelengths selected from NC spectra by RC, SR, SPA and CARS, respectively.

Method	Number of Optimal Wavelengths	Specific Wavelengths	Wavelength Reduction
RC	22	915, 924, 938, 950, 997, 1030, 1100, 1102, 1161, 1176, 1236, 1385, 1528, 1596, 1609, 1616, 1623, 1642, 1651, 1655, 1657, 1658 nm	94%
SR	15	915, 927, 960, 965, 1016, 1028, 1094, 1109, 1397, 1576, 1623, 1642, 1648, 1662, 1664 nm	96%
SPA	20	912, 915, 986, 1162, 1167, 1178, 1205, 1209, 1214, 1234, 1255, 1352, 1393, 1635, 1642, 1658,1660, 1662, 1664, 1667 nm	95%
CARS	11	924, 927, 936, 954, 1198, 1211, 1635, 1637, 1642, 1644, 1658 nm	97%

**Table 4:** Prediction of vitamin C (mg/100 g) in citrus peel by PLS model using optimal wavelengths.

Model	Number of Wavelength	Number of LV	Calibration		Cross-Validation		Prediction			ΔE
			*r*_C_	RMSE_C_	*r*_CV_	RMSE_CV_	*r*_P_	RMSE_P_	RPD	
RC-NC-PLS ^1^	22	9	0.936	16.316	0.908	19.887	0.901	20.354	2.855	4.038
SR-NC-PLS ^2^	15	10	0.942	15.432	0.937	16.418	0.936	16.689	3.482	1.257
SPA-NC-PLS ^3^	20	11	0.901	20.499	0.849	25.655	0.835	26.768	2.245	6.269
CARS-NC-PLS ^4^	11	7	0.814	24.494	0.785	25.767	0.787	25.916	2.358	1.422
RC-NC-MLR ^5^	22	-	0.913	19.148	0.852	25.112	0.848	26.776	2.319	7.628
SR-NC-MLR ^6^	15	-	0.955	13.430	0.948	14.651	0.949	14.814	4.260	1.384
SPA-NC-MLR ^7^	20	-	0.910	19.447	0.855	24.865	0.829	27.326	2.346	7.879
CARS-NC-MLR ^8^	11	-	0.842	23.578	0.836	26.663	0.818	29.409	2.214	5.831

^1^ PLS model built with optimal wavelengths selected from NC spectra by RC method; ^2^ PLS model built with optimal wavelengths selected from NC spectra by SR method; ^3^ PLS model built with optimal wavelengths selected from NC spectra by SPA method; ^4^ PLS model built with optimal wavelengths selected from NC spectra by CARS method; ^5^ MLR model built with optimal wavelengths selected from NC spectra by RC method; ^6^ MLR model built with optimal wavelengths selected from NC spectra by SR method; ^7^ MLR model built with optimal wavelengths selected from NC spectra by SPA method; ^8^ MLR model built with optimal wavelengths selected from NC spectra by CARS method.

**Table 5:** *F*-test and *t*-test double sample analysis of variance.

Model	Double Sample Analysis	Index	Predicted Value	Measured Value
SR-NC-MLR	*F*-test	average	149.861	150.334
		variance	3478.621	3464.791
		observed value	83	83
		d*f*	82	82
		*F*	1.004	
		*P* (F <= f) one-tailed	0.493	
		*F* (one-tailed critical value)	1.441	
	*t*-test	average	149.861	150.334
		variance	3478.621	3464.791
		observed value	83	83
		pooled variance	3471.706	
		assumed mean difference	0	
		d*f*	164	
		*t* Stat	0.0517	
		*P* (*T* <= *t*) one-tailed	0.479	
		*t* (one-tailed critical value)	1.654	
		*P* (*T* <= *t*) two-tailed	0.959	
		*t* (two-tailed critical value)	1.975	

## Data Availability

Data sharing not applicable.

## References

[1] El-OtmaniA M., Ait-Oubahou A., Zacarías L., Yahia E.M. (2011). Citrus *spp*.: Orange, mandarin, tangerine, clementine, grapefruit, pomelo, lemon and lime. Postharvest Biology and Technology of Tropical and Subtropical Fruits.

[2] Li B.B., Smith B., Hossain M.M. (2006). Extraction of phenolics from citrus peels I. Solvent extraction method. Sep. Purif. Technol..

[3] Rafiq S., Kaul R., Sofi S.A., Bashir N., Nazir F., Nayik G.A. (2018). Citrus peel as a source of functional ingredient: A review. J. Saudi Soc. Agr. Sci..

[4] Mamma D., Christakopoulos P. (2008). Citrus peels: An excellent raw material for the bioconversion into value-added products. Tree Forest. Sci. Biotechnol..

[5] Nair S.A., SR R.K., Nair A.S., Baby S. (2018). Citrus peels prevent cancer. Phytomedicine.

[6] Rehman Z.U. (2006). Citrus peel extract—A natural source of antioxidant. Food Chem..

[7] Güzel M., Akpınar Ö. (2019). Production and characterization of bacterial cellulose from citrus peels. Waste Biomass. Valor..

[8] Dhillon S.S., Gill R.K., Gill S.S., Singh M. (2004). Studies on the utilization of citrus peel for pectinase production using fungus *Aspergillus niger*. Int. J. Environ. Stud..

[9] Indulekha J., Gokul Siddarth M.S., Kalaichelvi P., Arunagiri A., Mohan B.R., Srinikethan G., Meikap B. (2017). Characterization of citrus peels for bioethanol production. Materials, Energy and Environment Engineering.

[10] Sir Elkhatim K.A., Elagib R.A.A., Hassan A.B. (2018). Content of phenolic compounds and vitamin C and antioxidant activity in wasted parts of Sudanese citrus fruits. Food Sci. Nutr..

[11] Mditshwa A., Magwaza L.S., Tesfay S.Z., Opara U.L. (2017). Postharvest factors affecting vitamin C content of citrus fruits: A review. Sci. Hortic..

[12] Podmore I.D., Griffiths H.R., Herbert K.E., Mistry N., Mistry P., Lunec J. (1998). Vitamin C exhibits pro-oxidant properties. Nature.

[13] Miller P., Ebert B. (2017). Vitamin C regulates stem cells and cancer. Nature.

[14] Bozkir H., Tekgül Y., Erten E.S. (2021). Effects of tray drying, vacuum infrared drying, and vacuum microwave drying techniques on quality characteristics and aroma profile of orange peels. J. Food Process Eng..

[15] Suri S., Singh A., Nema P.K. (2021). Recent advances in valorization of citrus fruits processing waste: A way forward towards environmental sustainability. Food Sci. Biotechnol..

[16] Nielsen S.S., Nielsen S.S. (2010). Vitamin C determination by indophenol method. Food Analysis Laboratory Manual.

[17] Card D.J., Harrington D. (2019). Methods for assessment of vitamin C. Laboratory Assessment of Vitamin Status.

[18] Yusuf E., Tkacz K., Turkiewicz I.P., Wojdyło A., Nowicka P. (2021). Analysis of chemical compounds’ content in different varieties of carrots, including qualification and quantification of sugars, organic acids, minerals, and bioactive compounds by UPLC. Eur. Food Res. Technol..

[19] El-Hawiet A., Elessawy F.M., El Demellawy M.A., El-Yazbi A.F. (2022). Green fast and simple UPLC-ESI-MRM/MS method for determination of trace water-soluble vitamins in honey: Greenness assessment using GAPI and analytical eco-scale. Microchem. J..

[20] Pisoschi A.M., Pop A., Serban A.I., Fafaneata C. (2014). Electrochemical methods for ascorbic acid determination. Electrochim. Acta..

[21] Antonelli M.L., D’Ascenzo G., Laganà A., Pusceddu P. (2002). Food analyses: A new calorimetric method for ascorbic acid (vitamin C) determination. Talanta.

[22] Pisoschi A.M., Danet A.F., Kalinowski S. (2008). Ascorbic acid determination in commercial fruit juice samples by cyclic voltammetry. J. Autom. Methods Manag. Chem..

[23] Shekhovtsova T.N., Muginova S.V., Luchinina J.A., Galimova A.Z. (2006). Enzymatic methods in food analysis: Determination of ascorbic acid. Anal. Chim. Acta.

[24] Herbig A.L., Renard C.M.G.C. (2017). Factors that impact the stability of vitamin C at intermediate temperatures in a food matrix. Food Chem..

[25] He H.J., Wang Y., Zhang M., Wang Y., Ou X., Guo J. (2022). Rapid determination of reducing sugar content in sweet potatoes using NIR spectra. J. Food Compos. Anal..

[26] Kutsanedzie F.Y.H., Guo Z., Chen Q. (2019). Advances in nondestructive methods for meat quality and safety monitoring. Food Rev. Int..

[27] Chen Q., Lin H., Zhao J., Chen Q., Lin H., Zhao J. (2021). Near-Infrared Spectroscopy Technology in Food. Advanced Nondestructive Detection Technologies in Food.

[28] Miseo E.V., Meyer F., Ryan J., Crocombe R., Leary P., Kammrath B. (2021). Portable Near-Infrared Spectroscopy in Food Analysis. Portable Spectroscopy and Spectrometry.

[29] He H.J., Wang Y., Ou X., Ma H., Liu H., Yan J. (2023). Rapid determination of chemical compositions in chicken flesh by mining hyperspectral data. J. Food Compos. Anal..

[30] Tirado-Kulieva V.A., Hernández-Martínez E., Suomela J.-P. (2022). Non-destructive assessment of vitamin C in foods: A review of the main fndings and limitations of vibrational spectroscopic techniques. Eur. Food Res. Technol..

[31] Valentini M., dos Santos G.B., Vieira B.M. (2021). Multiple linear regression analysis (MLR) applied for modeling a new WQI equation for monitoring the water quality of Mirim Lagoon, in the state of Rio Grande do Sul-Brazil. SN Appl. Sci..

[32] Albergaria J.T., Martins F.G., Alvim-Ferraz M.C.M., Delerue-Matos C. (2014). Multiple linear regression and artificial neural networks to predict time and efficiency of soil vapor extraction. Water Air Soil Poll..

[33] Mohammadi-Moghaddam T., Razavi S., Sazgarnia A., Taghizadeh M. (2018). Predicting the moisture content and textural characteristics of roasted pistachio kernels using Vis/NIR reflectance spectroscopy and PLSR analysis. J. Food Meas. Charact..

[34] Kokalj M., Rihtarič M., Kreft S. (2011). Commonly applied smoothing of IR spectra showed unappropriate for the identification of plant leaf samples. Chemom. Intell. Lab. Syst..

[35] Rinnan Å., van den Berg F., Engelsen S.B. (2009). Review of the most common pre-processing techniques for near-infrared spectra. TrAC-Trends Anal. Chem..

[36] Peng J., Peng S., Jiang A., Wei J., Li C., Tan J. (2010). Asymmetric least squares for multiple spectra baseline correction. Anal. Chim. Acta.

[37] Roy I.G. (2015). On computing first and second order derivative spectra. J. Comput. Phys..

[38] Gautam R., Vanga S., Ariese F., Umapathy S. (2015). Review of multidimensional data processing approaches for Raman and infrared spectroscopy. EPJ Techn. Instrum..

[39] Jiao Y., Li Z., Chen X., Fei S. (2020). Preprocessing methods for near-infrared spectrum calibration. J. Chemom..

[40] Oliveri P., Malegori C., Simonetti R., Casale M. (2019). The Impact of Signal Pre-Processing on the Final Interpretation of Analytical Outcomes—A tutorial. Anal. Chim. Acta.

[41] AOAC (2007). Method 967.21: Vitamin C (total) in food. Official Methods of Analysis of the Association of Official Analytical Chemists.

[42] Gatius F., Miralbés C., David C., Puy J. (2017). Comparison of CCA and PLS to explore and model NIR data. Chemom. Intell. Lab. Syst..

[43] Li B., Martin E., Morris J. (2001). Latent variable selection in partial least squares modelling. IFAC Proc. Vol..

[44] Gowen A.A., Downey G., Esquerre C., O’Donnell C.P. (2011). Preventing over-fitting in PLS calibration models of near-infrared (NIR) spectroscopy data using regression coefficients. J. Chemometr..

[45] Wang Y.Y., He H.J., Jiang S.Q., Ma H.J. (2022). Nondestructive determination of IMP content in chilled chicken based on hyperspectral data combined with chemometrics. Int. J. Agr. Biol. Eng..

[46] Kasemsumran S., Du Y.P., Li B.Y., Maruo K., Ozaki Y. (2006). Moving window cross validation: A new cross validation method for the selection of a rational number of components in a partial least squares calibration model. Analyst.

[47] Jiang S.Q., He H.J., Ma H.J., Chen F.S., Xu B.C., Liu H., Zhu M.M., Kang Z.L., Zhao S.M. (2021). Quick assessment of chicken spoilage based on hyperspectral NIR spectra combined with partial least squares regression. Int. J. Agr. Biol. Eng..

[48] He H.J., Wu D., Sun D.W. (2015). Nondestructive spectroscopic and imaging techniques for quality evaluation and assessment of fish and fish products. Crit. Rev. Food Sci..

[49] Zou X., Zhao J., Malcolm J.W.P., Mel H., Mao H. (2010). Variables selection methods in near-infrared spectroscopy. Anal. Chim. Acta.

[50] Zhu Y.D., He H.J., Jiang S.Q., Ma H.J., Chen F.S., Xu B.C., Liu H., Zhu M.M., Zhao S.M., Kang Z.L. (2021). Mining hyperspectral data for non-destructive and rapid prediction of nitrite content in ham sausages. Int. J. Agr. Biol. Eng..

[51] Mehmood T., Liland K.H., Snipen L., Sæbø S. (2012). A review of variable selection methods in Partial Least Squares Regression. Chemom. Intell. Lab. Syst..

[52] Soares S.F.C., Gomes A.A., Araujo M.C.U., Filho A.R.G., Galvão R.K.H. (2013). The successive projections algorithm. TrAC-Trends Anal. Chem..

[53] Zheng K., Li Q., Wang J., Geng J., Cao P., Sui T., Wang X., Du Y. (2012). Stability competitive adaptive reweighted sampling (SCARS) and its applications to multivariate calibration of NIR spectra. Chemom. Intell. Lab. Syst..

[54] Glatting G., Kletting P., Reske S.N., Hohl K., Ring C. (2007). Choosing the informative fit function: Comparison of the Akaike information criterion and the F-test. Med. Phys..

[55] Adusah A.K., Brooks G.P. (2011). Type I error inflation of the separate-variances welch t test with very small sample sizes when assumptions are met. J. Mod. Appl. Stat. Meth..

